# Cognitive Status Classification Among Older Adults: A Study from SHARE-HCAP

**DOI:** 10.3390/jcm14248625

**Published:** 2025-12-05

**Authors:** Aitana Sanz, Laura Galiana, Irene Fernández

**Affiliations:** Department of Methodology of the Behavioural Sciences, University of Valencia, 46010 Valencia, Spain; aisanzde@alumni.uv.es (A.S.); laura.galiana@uv.es (L.G.)

**Keywords:** cognitive impairment, risk factors, protective factors, odds, logistic regression

## Abstract

**Background:** Cognitive impairment is a major health problem, so several studies worldwide have studied its aggravating and protective factors. However, few studies have looked into the prediction of better or worsening cognitive status based on the presence of the most relevant biopsychosocial factors. Thus, the aim of this study is to predict cognitive status classification, specifically into normal cognitive status, mild cognitive impairment, and severe cognitive impairment, based on the most studied risk and protective factors in the context of the Survey of Health, Ageing and Retirement in Europe and Harmonized Cognitive Assessment Protocol association (SHARE-HCAP) project. **Methods:** Participants were from five European countries, and measures included their cognitive status classification from SHARE-HCAP and several associated factors previously measured in the eighth wave of SHARE. **Results:** A multinomial logistic regression was performed, with normal cognition as the reference category. Most individuals were correctly classified. **Conclusions:** Frequent participation in social activities and good cognitive task performance were associated with a lower likelihood of mild cognitive impairment compared to normal cognitive status. In turn, higher scores in depression and social network were associated with an increased likelihood of being classified as MCI in contrast to normal cognition. Additionally, being a woman and having worse cognitive performance were predictors of severe cognitive impairment classification over normal cognition status. Depression also contributed to a higher likelihood of being classified as mild and severe cognitive impairment, in contrast to a normal cognitive status. These findings highlight the importance of preventive medical check-ups and interventions before the onset of the first cognitive decline symptoms.

## 1. Introduction

Recent data indicate that the number of people over 60 years of age will continue to grow over time, resulting in the ageing of the population globally [1,2]. This could imply that the prevalence of conditions and difficulties associated with ageing will also increase, which will very likely have an impact on the prevalence of neurocognitive disorders, such as dementia.

Nowadays, older adults’ cognitive function is studied from a holistic perspective. From a biopsychosocial approach, all facets that accompany ageing can be integrated to optimize the ageing process and to achieve successful ageing (SA). This model entails biological, psychological, and social aspects, as well as the relationship among them [3]. In this line, two popular models were developed to capture the process of ageing successfully: the model of Selection Optimization and Compensation (SOC) [4] and the Comprehensive Preventive Corrective Proactive model (Comprehensive PCP) [5].

The SOC model [4] considers the adaptive nature of the ageing process. This model aims to understand the regulation process through different levels of analysis, where life management is mediated by three main components. These are selection, used to set goals and direct development; optimization, to acquire, invest, apply, and refine relevant and desirable outcomes; and compensation, where new alternative and compensatory behaviours are used to maintain levels of functioning to face losses and decreases in personal resources. This model has been tested in older adults, and the results indicated that middle-aged adults used the SOC components more often than in late adulthood, where declining biological resources limit these regulatory processes [6].

In turn, the Comprehensive PCP model [5] has been widely recognized due to its conceptualization of SA. In this model, the main assumption is that increased age involves a series of health- and socially related stress factors associated with ageing. There are two types of stressors: normative stressors, related to events that normally take place in people’s lives, and accumulated stressors, individualized and diverse events that occasionally occur. To face and reduce them, the model posits that individuals ought to behave proactively to adapt. This goal-directed behavioural orientation is used for preventive and corrective aims, as it improves health and addresses stressors. After the appearance of stressors, it activates resources and compensatory activities correspondingly. Additionally, the model considers contextual influences, such as the individual’s social status, demographic characteristics, environmental context, and living conditions. The Comprehensive PCP model conceptualizes SA both as a process and as an outcome. The outcomes of SA include quality of life (QoL), life satisfaction, meaning in life, and maintenance of activities and relationships. Finally, the model is dynamic, implying that every element interacts and influences each other.

Within this theoretical framework, cognitive function can be considered a normative stressor in old age. Its deterioration over time, particularly noticeable from middle age onwards, can affect older adults’ QoL [7]. Therefore, this is an essential reason for preventing its malfunctioning.

Cognitive impairment is marked by a progression from a preclinical state, where symptoms are not visible, to an overt state of dementia, which, in some cases, occurs prematurely. Nevertheless, moderate declines in cognition take place before dementia. Mild cognitive impairment (MCI) is a transitional stage between normal cognitive functioning and cognitive impairment or dementia, where the decline exceeds the normative decline associated with an individual’s age and level of education [8,9,10,11]. Individuals with MCI complain about subjective memory problems, but they can still perform their daily activities. It is estimated that around 15% of individuals with MCI develop dementia every year, and one out of three people with MCI ends up with a diagnosis of Alzheimer’s disease (AD) within a 5-year period [10,12,13]. Despite not being a condition for developing dementia, individuals displaying MCI are at a higher risk of developing it. Conversely, MCI may revert to normal cognition, and many consider it to be an optimal stage for early detection and intervention of cognitive impairment [9,10,11,12,13,14]. Some studies have estimated its global prevalence at 15.56% [8]; however, many others have estimated it to be 19% in people aged 50 years or older [13,14,15]. Regarding dementia, it has been rated by the WHO ranking as the seventh cause of death globally, with women being disproportionately affected [16]. Dementia is caused by damage to brain cells that ultimately causes brain cell death, which leads to a decline in cognitive functioning. This impairment includes cognitive domains, such as perception, attention, learning and memory, language, executive function, and social recognition; however, it also affects an individual’s affective and behavioural areas [11,17,18].

Several factors have been shown to have an influence on the development of MCI and dementia. These are classified as non-modifiable risk factors, modifiable risk factors, and protective factors. Non-modifiable risk factors encompass older age, gender, genetics (including biomarkers and the APOE4 allele presence) and family history [12,19,20,21,22]. Modifiable risk factors, in turn, consist of chronic and cardiovascular diseases (such as hypertension, diabetes mellitus, obesity, hyperlipidaemia, chronic pain, or hearing impairment), glaucoma, smoking, excessive alcohol consumption, unhealthy dietary habits, physical inactivity, stress, depression, anxiety, traumatic brain injury, migraine, air pollution, loneliness, or social isolation [12,14,19,20,21,22,23,24]. All the aforementioned factors contribute to chronic inflammation or neuroinflammation [21], which can be explained by two popular theories about cognitive impairment. The Inflammation Hypothesis [25] suggests that chronic inflammatory processes are due to ageing, which deteriorates the biological system. Furthermore, the Vascular Depression Hypothesis [26] proposes that neurovascular deterioration accelerates the appearance of depressive symptomatology in old age.

In turn, protective factors include a high educational level, cognitive stimulation, socioeconomic position, and social support networks [12,14,16,20,21,22,27]. These factors can be included in and contribute to an evidence-based concept that compensates for the losses of cognitive decline. The cognitive reserve theory [28] defines cognitive reserve as the capacity of the brain to optimize, be efficient, and tolerate brain damage, delaying the clinical expression of cognitive and behavioural symptoms. Cognitive reserve is also considered a protective factor against cognitive impairment [9,29].

Several studies have employed inferential and multivariate techniques to study the risk factors for dementia. For example, Khondoker et al. [23] used a clustering method to create diverse multimorbidity groups and then associated them with dementia. Their findings showed that mental health and cardiometabolic clusters were twofold more likely to develop dementia. The inflammatory cluster was also associated with dementia, but with an intermediate effect. In turn, Kim [20] used LASSO regression to distributionally predict cognitive function based on risk factors, associating them with cognitive impairment and predicting its long-term impact. The author found that older age, low educational level, few social interactions, and deteriorated health status were risk factors for cognitive impairment. Finally, Marselli et al.’s [29] results suggested that cognitive reserve is a protective factor against cognitive decline and MCI, favouring the regression from MCI to normal cognition.

The present study aims to predict the classification odds for different cognitive statuses (normal, MCI, and severe cognitive impairment [SCI]) according to participants’ previous scores for the most relevant risk and protective factors documented in the literature. The tested hypotheses are, firstly, that the probability of developing MCI will be affected at least by one of the most cited and predictive factors (sociodemographic, health conditions, including physical and mental health, lifestyle, social relationships, or cognitive function). Secondly, the probability of developing SCI will be affected at least by one of the most cited and predictive factors in the scientific literature (sociodemographic, health conditions, social relationships, or cognitive function).

## 2. Materials and Methods

### 2.1. Sample and Procedure

The data were obtained from the Survey of Health, Ageing and Retirement in Europe (SHARE) [30] and the SHARE–Harmonized Cognitive Assessment Protocol (SHARE-HCAP) [31]. SHARE is a longitudinal panel study created in 2004 to study people’s health, socioeconomic status and social networks. The project is aimed at respondents aged 50 and older across 27 European countries and Israel. Since 2004, nine waves of data have been released. SHARE-HCAP is an associated dataset developed within an agreement to harmonize cognitive measurements in a global network of studies. SHARE-HCAP represents the standardized assessment of cognition in Europe. However, only five European countries participated in the first wave of SHARE-HCAP: the Czech Republic, Denmark, France, Germany, and Italy.

Regarding the sampling strategy, the SHARE project usually follows a probabilistic approach, with slight variations across different countries. The work by Bethmann et al. [32] provides additional details about the sampling strategy used.

The present study used data from wave eight, as well as information about the cognitive status of participants extracted from SHARE-HCAP. All individuals who reported having Alzheimer’s/dementia/senility were excluded from the analyses. The final sample was composed of individuals who participated in wave 8 and in SHARE-HCAP, with a total of 2109 participants. Out of them, 45.7% were men and 54.3% were women, with an average age of 73.57 years (SD = 7.48), ranging between 61 years old and 99 years old. There were 5 European countries represented in the sample: Germany (22.7%), Italy (14.7%), France (22.2%), Denmark (21.3%), and the Czech Republic (19.2%).

### 2.2. Instruments

The predictive variables included data on sociodemographic aspects, risk behaviours, physical and mental health, social networks, and cognitive function, measured during SHARE wave 8. The outcome variable was the classification of individuals’ cognitive status from SHARE-HCAP, measured approximately two years after data were gathered in SHARE wave 8.

The sociodemographic data included age at the moment of interview in wave 8, gender (0 = female, 1 = male), country, and educational level. For education, the 1997 version of the International Standard Classification of Education (ISCED-97) [33] was used to code the participants’ educational levels. Seven levels of education were considered as follows: 0 = pre-primary, 1 = first stage of basic education, 2 = second stage of basic education, 3 = upper secondary education, 4 = post-secondary non-tertiary education, 5 = first stage of tertiary education, and 6 = second level of tertiary education.

The measures of behavioural risks were units of alcohol intake in the previous 7 days, smoking behaviour (0 = non-smoker; 1 = past smoker; 2 = smoker), and physical inactivity. Physical inactivity was measured with an item asking how frequently the respondent performed vigorous sports or activities, coded as 1 = “more than once a week”, 2 = “once a week”, 3 = “one to three times a month”, and 4 = “hardly ever or never”.

Regarding physical health, we considered whether the respondent had ever been diagnosed with the following chronic disorders: heart attack, stroke, hypertension, hypercholesterolemia, and diabetes. For each disorder, the responses were coded dichotomously (1 = Yes, 0 = No).

The measures of mental health included depression, quality of life (QoL), and social and intellectual activity participation. Depression was measured with the Euro-D [34], a screening scale for depressive symptomatology that assesses the presence of the following 12 symptoms: depressed mood, pessimism, suicidal ideation, guilt, sleep, lack of interest, irritability, lack of appetite, fatigue, concentration problems, lack of enjoyment, and crying. Scores range from 0 = “not depressed” to 12 = “very depressed”. Activity participation was differentiated into social or intellectual. Social activity participation included volunteerism, attending educational courses, attending sports/social clubs, and taking part in political/community-related organizations. Intellectual activity participation included reading books/magazines/newspapers, completing word/number games, and playing chess/cards/similar. For each activity, one point was scored if the participant reported performing that activity within the last 12 months, and zero points were recorded otherwise. For each type of activity participation, the final score was the sum of the activities reported by the individual. Finally, QoL was assessed using the CASP-12, a modified version of the CASP-19 [35]. This scale contains 12 items of 4 sub-scales (control, autonomy, self-realisation, and pleasure). Items are scored on a four-point Likert scale, rated as 1 = “never”, 2 = “rarely”, 3 = “sometimes”, and 4 = “often”, with high scores interpreted as high QoL.

Regarding social relationships, loneliness was measured by the Three-Item Loneliness scale [36], a short version of the R-UCLA Loneliness scale [37,38]. Items were answered on a three-point Likert scale (1 = “hardly ever or never”, 2 = “some of the time”, and 3 = “often”); the minimum score is 3 = “not lonely”, and the maximum score is 9 = “very lonely”. Regarding social networks, we used the Social Network Index (SNI). To create it, we followed the procedure described in Torres et al. [39]. The scale taps five characteristics of social networks: network size, proximity, frequency of contact, degree of emotional support, and network diversity. The final score ranges from 0 to 20.

For cognitive function assessment, we used indicators of memory, orientation, numeracy, and verbal fluency. Memory was differentiated into recent and delayed recall using the 10-Word Recall Test. The task consisted of remembering a list of 10 words immediately and after a short period of time. The score represents the total number of words recalled at each time. For orientation, the respondents were asked four questions about the date, day of the week, month, and year. The score ranges between 0 and 4, with the highest score for the most oriented respondents. Regarding numeracy, the respondents’ mathematical performance was measured by five items related to subtraction calculation. The numeracy score is based on correct subtractions and ranges between 0 and 5, with higher scores representing better performance. Finally, verbal fluency was measured by asking the participants to report all animals they could think of in a period of 60 s. The score represents the number of reported animals.

Finally, cognitive status was classified as 1 = “normal”, 2 = “mild cognitive impairment”, and 3 = “severe cognitive impairment”. This indicator is provided in the SHARE-HCAP dataset; further details on the procedure followed to derive it are described in Börsch-Supan et al. [40].

### 2.3. Statistical Analysis

First, descriptive statistics of all variables included in the model were computed. For nominal and ordinal variables, frequency distributions were extracted. For quantitative variables, means and standard deviations were calculated. Next, the assumption of proportional odds for ordinal regression was tested using the contrast of parallel lines. Results with an associated *p*-value greater than the critical value 0.05 indicated that the assumption of proportional odds was met by the data, and ordinal regression could be computed. Otherwise, the odds were not proportional and nominal regression was computed instead. Finally, the regression model was specified, in which the dependent variable was the respondents’ cognitive status, and the independent variables were age, gender, education, alcohol and smoking behaviours, physical inactivity, heart attack, stroke, hypertension, hypercholesterolemia, diabetes, depression, QoL, social networks, loneliness, social and intellectual activity participation, recent and delayed recall, orientation, numeracy, and verbal fluency. To assess model fit, we employed the likelihood ratio test statistic (*G*^2^), Nagelkerke’s pseudo-*R*^2^, and the deviance statistic. The *G*^2^ statistic compares the proposed model against the null model, with *p*-values less than 0.05 indicating a significantly improved fit of the proposed model. Nagelkerke’s *R*^2^ reports the proportional reduction in the deviance. Finally, the deviance statistic contrasts the equality between the proposed model and the saturated model, with *p*-values greater than 0.05 indicating that the proposed model does not differentiate from the saturated model. Effects of the independent variables were interpreted in terms of odds ratios. All analyses were performed in SPSS 28 [41].

## 3. Results

### 3.1. Sample Characteristics

The descriptive statistics of the variables employed in this study are available in Table 1. There were slightly more female individuals in the sample. The average age was 73.57 years old (SD = 7.48), with a minimum age of 61 and a maximum of 99. Also, the most frequent educational level documented by the participants was “upper secondary education” and “first stage of tertiary education” (37.8% and 25.7%, respectively).

The descriptive statistics showed that about half of the participants were non-smokers (55.9%), were physically inactive (49.8% hardly ever or never engaged in vigorous sports or activities), suffered from hypertension (49.9%), and did not suffer from heart attack, hypercholesterolemia, stroke, or diabetes. In addition, the participants engaged in more intellectual than social activities.

Regarding levels of mental health, the mean score in quality of life was 38.29. As it was measured with the CASP-12, and the scale ranges from 12 to 48, these levels can be considered high. The mean in depression, in turn, was 2.34. As it was measured with the EURO-D, which ranges from 0 to 12, this score can be considered low. Along the same line, the mean in loneliness was 3.97. As loneliness was measured with the three-item version of the R-UCLA Loneliness scales, in which scores range from 3 to 9, 3.97 can also be considered low. Finally, regarding social networks, the levels can be considered medium, as the mean value was 9.61, and it was measured with the Social Network Index, which ranges from 0 to 20.

### 3.2. Regression Analyses

To predict the odds of classification for the different cognitive states (normal, MCI, and SCI), a multinomial logistic regression was performed. Given the high number of predictors included, a backward step-wise variable selection approach was used. All the independent variables were included in the equation and sequentially removed in order to meet the removal criteria, with the smallest effect on the dependent variable being considered first for removal. A total of 11 independent variables were dropped from the analysis: hypertension, hypercholesterolemia, smoking, heart attack, vigorous activities, diabetes, intellectual activities, loneliness, stroke, QoL, and alcohol use. The variables included in the model were gender, educational level, age, depression, social activities, social network, immediate recall, delayed recall, orientation, numeracy, and verbal fluency. The likelihood ratio test results for all initial predictors are available in Table 2. The overall model fit was *G*^2^(32) = 633.04 (*p* < 0.001), rejecting the null hypothesis. Moreover, Nagelkerke’s *R^2^* value was 0.365, indicating a 36.5% reduction in deviance. Lastly, the deviance statistic was χ^2^(3552) = 2386.23 (*p* = 1.00), indicating that the proposed model does not significantly differ from the saturated model.

The parameter estimates are available in Table 3. The reference category of the outcome variable was normal cognitive function. Among all the variables that were statistically significant in the overall model, only some of them were statistically significant predictors of MCI, as compared to normal cognition. Some of these were social activity participation, immediate recall, delayed recall, numeracy, and verbal fluency. Specifically, an increase of 1 point on the scale of social activity participation decreased the odds of being classified as MCI by 1.26 (1/0.79) times. Similarly, the odds of being classified as MCI decreased 1.14 (1/0.88) times for a 1-point increase in immediate recall. An increase of 1 point in delayed recall decreased the probability of being classified as MCI by 1.25 (1/0.80) times. These relations were lower for numeracy and verbal fluency: an increase of 1 point in the numeracy score only decreased the odds of being classified as MCI by 1.11 (1/0.90), whereas an increase of 1 point in verbal fluency only produced a decrease of 1.05 (1/0.95). Additionally, the rest of the statistically significant predictors were depression and social network, with higher scores being associated with an increased likelihood of being classified as MCI in contrast to normal cognition. That is, an increase of 1 point on the scale of depression produced an increase of 1.15 in the odds of being classified as MCI in comparison to normal cognition. In contrast, an increase of 1 point on the social network scale produced an increase of 1.05 in the odds of being classified as MCI.

Regarding the parameter estimates for SCI, the statistically significant variables were gender, delayed recall, orientation, numeracy, verbal fluency, and depression. Being male was associated with a decrease of 1.59 (1/0.63) in the likelihood of being classified as SCI in contrast to normal cognition. An increase of 1 point in delayed recall also produced a decrease of 1.67 (1/0.60) in the odds of suffering from SCI. Along the same line, a 1-point increase in orientation reduced the odds of suffering from SCI by 1.85 (1/0.54) times. And the odds of being classified as SCI decreased 1.23 (1/0.81) and 1.18 (1/0.85) times with a 1-point increase in numeracy and verbal fluency, respectively. In turn, higher scores for depression were associated with an increased likelihood of being classified as SCI, specifically, an increase of 1 point (one symptom) on the scale of depression increased the odds of suffering from SCI by 1.19.

Finally, a prediction of the nominal regression was performed to classify the subjects in this study. The cases correctly predicted by the model in each category were 92.3% (*n* = 1091) of correct classification for normal cognition, 23.7% (*n* = 105) for MCI, and 38.1% (*n* = 64) for SCI. The total number of correctly classified cases was 70.3% (*n* = 1260).

## 4. Discussion

The ageing of the population is a fact worldwide [1,2], and a lot of studies warn about an increase in difficulties and conditions associated with it, such as health and mental problems or disabilities [2,42,43]. Among all of them, cognitive impairment is a major concern due to its global impact and prevalence among older adults; indeed, it is one of the main causes of dependence and mortality [11,16]. Some studies have tried to explain cognitive impairment occurrence during the ageing process through theoretical models [3,4,5,25,26,28], its risk or protective factors [12,16,21,22,27], its implications on people’s QoL [7], and its prediction [20,23,24,29]. In this scenario, the aim of this study was to examine and predict the classification odds of cognitive status in older adults according to the most documented factors in the literature using data from SHARE, a population-based study carried out in Europe, linking it with recently released data on cognitive status classification for a subset of the participating sample: the SHARE-HCAP sub-study.

Our findings showed that most of the sample, specifically, 70.3% of the individuals, were correctly classified by the estimated model for each cognition status. Overall, 92.3% of the normal cognitive status category was correctly classified, whereas this dropped to 38.1% for SCI and to 23.7% for MCI. Although the proportion of correctly classified cases in general was adequate, the model showed lower proportions of correctly classified individuals in the mildly and severely impaired groups, as compared to the correct classification of individuals in the normal cognition status group. Additional predictive markers, such as biomarkers, could improve sensitivity for early-stage impairment detection. Regarding the factors that determined this classification, evidence for both hypotheses was found. Several variables were selected and included in the model due to their significant effect on cognitive status prediction, but it was found that not all of them contributed equally to the classification.

Relevant variables for the classification of the respondents’ cognitive status were those related to sociodemographic data and cognitive function. In addition, variables related to mental health and social relationships, such as depression, social activities, and social networks, also proved to be relevant predictors of cognitive status. Regarding MCI, the results were in line with the first hypothesis and the existing literature; participating in multiple social activities and displaying good performance on cognitive tasks are protective factors that can prevent or delay the development of MCI [44,45,46,47]. Conversely, MCI was more likely to occur when levels of depression were high, thus reinforcing the literature that identifies depression as a risk for a mild stage of cognitive impairment [24,44]. Regarding previous insights on the role of social activity, some controversial outcomes have been gathered. Although social activity has been traditionally considered a protective factor due to social contact, in some studies, it showed no effect in preventing dementia [48]. In some other studies, social activity did show an impact on overall global cognition, but not in specific cognitive domains [49]. Regarding social networks, although considered a protective factor [20], the results of this study suggest that social activity participation protects against MCI, but broader and richer social networks were associated with an increased likelihood of being classified as MCI and SCI, in contrast to normal cognition. A possible explanation could be that as a person’s dependency level increases, their closest social environment looks after them more frequently. Eventually, the older adult ends up living with their family [12].

The results for SCI were also in line with the second hypothesis and the existing literature. Namely, being a man and displaying good cognitive performance were associated with a decreased probability of being classified as SCI [19,45,46]. Conversely, the respondents were more likely classified as SCI when they were women and had high levels of depression [21,44,50]. The contribution of gender to SCI development was more expected than its contribution to MCI, specifically for females. Oestrogens during the menstrual cycle are neuroprotectors, but some studies suggest that a decrease in their levels can be related to cognitive impairment. Concretely, in Rahman et al. [51], the menopause transition was the major female-specific risk factor for Alzheimer’s disease, where a decline in oestrogens was involved in biomarker abnormalities. Also, in Yoo et al. [52], long fertility periods (when oestrogens are activated) reduced the risk of dementia, while having later menstruation and earlier menopause had the opposite effect. What is more, both linked their molecular actions to inflammatory brain effects, as the link between neuroinflammation, cognitive impairment, and ageing is a well-established fact [21,25]. However, regarding MCI and SCI outcomes, several differences have been found between them. While social activities and social networks had a significant effect on MCI, they had no impact on SCI, for which gender and orientation were more important. This could confirm the operational window or optimal stage approach to intervene on MCI [9,10,11,12,13,14]. Activities related to social connections and cognitive stimulation could stop or reverse an individual’s MCI status to a normal status, contributing to cognitive reserve, possibly by reinforcing existing neuronal connections or even creating new ones [9].

On the contrary, some of the variables considered had no effects on MCI or SCI classification versus the normal state. These variables were related to physical health and lifestyle, intellectual activities, QoL, and loneliness. These results contrast with the existing literature, where these variables are identified as relevant correlates of cognitive impairment. For example, in Trevisan et al. [24], the probability of progression from MCI to a worse cognitive status was related to a high number of diseases (multimorbidity), but not to specific diseases. In this line, the results of Khondoker et al. [23] suggested that mental health, cardiometabolic factors, inflammation, and cancer were risk factors for dementia. These results are especially remarkable because when chronic diseases are measured in clusters, studies report them as relevant predictors, whereas they are rarely identified as predictors when considered separately, as is the case with the current results. Regarding lifestyle factors, in Wang et al. [53], a healthy lifestyle decreased the risk of MCI and all types of dementia in people with or without cardiometabolic diseases. Also, a healthy lifestyle resulted in a modifier between air pollution and cognitive impairment for people with cardiometabolic diseases. Additionally, a lower QoL has been associated with cognitive impairment [7]; intellectual activities have been shown to reduce dementia risk in some studies [47,48]. Conversely, our loneliness and lifestyle outcomes were in line with Shen et al. [54] and Thorp et al. [55], respectively.

The unexpected results in this work, i.e., those inconsistent with the literature, are that age and educational level were statistically significant predictors but did not show a significant impact on MCI or SCI versus normal cognitive status classification. In many studies, advanced age is described as the most important and well-established risk factor [19,20,21,29], as well as a lower educational level [9,19,20,21,29]. Regarding age, Hajek et al. [19] indicated that age is considered a risk factor, but it may not be a factor itself; instead, it may be associated with overall health deterioration and immune system changes. Another possible explanation for our results could be the misconception of ageing: this natural condition does not necessarily lead to an overall deterioration [14]. In fact, the definition of ageing still generates debate [3], being explained as an accumulation of health and social stressors [5], an accumulation of pro-inflammatory agents or biological stressors [25], or as a continuous process of adaptation using three strategies to supply the loss of resources [4], among others, resulting in either SA or pathological ageing. Turning to educational level, despite most studies reporting its protective role, some explanations of our findings can be found in the literature. For example, Ma et al. [56] observed that educational attainment was only related to dementia risk when there was a high genetic risk (one or more APOE4 alleles).

### 4.1. Limitations and Strengths

This study had several limitations. Firstly, this study does not provide information about the change in variables over time, and it does not determine causality between them either. Secondly, the sample includes only 5 European countries from the 28 SHARE-participating countries because only 5 countries participated in SHARE-HCAP; therefore, the generalizability of results is limited. Another potential limitation of this study is selection bias. Although SHARE-HCAP employed strategies to oversample individuals with lower cognitive functioning and used informant interviews to collect data from participants unable to complete assessments [57], it remains possible that individuals with more severe cognitive impairment were underrepresented in the sample. This could lead to an underestimation of the prevalence or severity of cognitive impairment. Moreover, by using a backward step-wise variable selection approach, we assumed the consequence of leaving some conceptually relevant variables out of the equation [58]. In this vein, there are a series of potential confounders that were not included in the model, such as other clinical diagnoses that affect cognition. Given the already high number of predictors initially considered and the unavailability of these data, some potential confounding variables may have been excluded from the model. Finally, the cognitive information involved in the classification of cognitive status came from individuals’ performance on neuropsychological tests, as noted in Börsch-Supan et al. [40]. Therefore, the lack of sensitivity of such measures could lead to misclassification, particularly in distinguishing mild impairment from normal ageing. However, this study has many strengths, including the use of a large, heterogeneous sample of older adults from different European countries, obtained through probability-based sampling strategies. Additionally, there are many studies about the factors that influence the risk of cognitive decline, providing us with a basis on which to compare our results. Finally, our study provides the existing scientific literature with further evidence on the topic.

### 4.2. Future Perspectives

Considering the study outcomes together with previous evidence, cognitive impairment can be prevented by fostering the effects of protective factors and lessening the effects of risk factors. Performing routine physical and mental health screenings, such as blood draws, PET brain imaging (when there is suspected impairment), or psychological anamnesis for people aged 65 years or older, should be considered by public health systems. Also, intervention programs to prevent cognitive impairment should emphasize mental health, social connections, and cognitive training, recommended for everyone at any age, but especially for middle-aged adults, regardless of their health status, before the first symptoms of pathological ageing appear. These interventions would be welcomed, especially for European women, as they have shown greater probabilities of developing SCI. Finally, future research could extend the present work by incorporating samples from other countries and ethnic backgrounds to investigate cross-cultural differences in social activities, patterns of engagement, and their relationships with cognitive functioning among older adults. Such studies could help elucidate whether the protective role of social participation is consistent across diverse cultural and demographic settings.

## 5. Conclusions

The findings in this study indicated that cognitive status classification was determined by several factors. MCI was delayed by social activities and good performance on cognitive tasks but was aggravated by richer social networks and depression. Regarding SCI, being a woman, having worse performance on cognitive tasks, and depression increased its probability. Considering that this study was based on a European sample, these outcomes could suggest that earlier interventions over cognitive status are essential to prevent its impairment in this geographical area.

## Figures and Tables

**Table 1:** (**a**) Sociodemographic data from the final sample: categorical variables. (**b**) Sociodemographic data from final sample: continuous variables.

(a)	
Variable	*n* (%)
Gender (Male)	964 (45.7)
Educational level (ISCED-97 coding)	2097 (94.4)
None	67 (3.2)
Code 1: primary education	367 (17.4)
Code 2: lower secondary education	274 (13.0)
Code 3: upper secondary education	797 (37.8)
Code 4: post-secondary non-tertiary education	28 (1.3)
Code 5: first stage of tertiary education	542 (25.7)
Code 6: second stage of tertiary education	22 (1.0)
Missing data	12 (0.6)
Country	
Germany	478 (22.7)
Italy	309 (14.7)
France	468 (22.2)
Denmark	450 (21.3)
Czech Republic	404 (19.2)
Smoking behaviour	
Non-smoker	1178 (55.9)
Ex-smoker	693 (32.9)
Smoker	238 (11.3)
Vigorous sport or activities	
More than once a week	625 (29.6)
Once a week	288 (13.5)
One to three times a month	150 (7.1)
Hardly ever or never	1050 (49.8)
Heart attack (yes)	328 (15.6)
Hypertension (yes)	1053 (49.9)
Hypercholesterolemia (yes)	587 (27.8)
Stroke (yes)	93 (4.4)
Diabetes (yes)	346 (16.4)
(**b**)	
**Variable**	**M ± SD**
Age (wave 8)	73.57 ± 7.48
Alcohol intake (units)	4.80 ± 8.17
Verbal fluency	20.45 ± 7.51
Social activity participation	0.73 ± 0.91
Intellectual activity participation	1.66 ± 1.00
Social Network Index (SNI)	9.61 ± 3.48
Quality of life (QoL)	38.29 ± 6.10
Immediate recall	4.86 ± 1.77
Delayed recall	3.34 ± 2.19
Depression	2.34 ± 2.19
Loneliness	3.97 ± 1.38
Numeracy	4.07 ± 1.43

**Table 2:** Results of likelihood tests for multinomial regression predictors.

Predictor	−2LL	χ^2^	df	*p*
Removed from the model				
Hypertension	2362.64	0.13	2	0.936
Hypercholesterolemia	2362.76	0.13	2	0.938
Smoking	2363.64	0.87	4	0.929
Heart attack	2364.50	0.86	2	0.649
Vigorous activities	2369.12	4.62	6	0.594
Diabetes	2370.03	0.91	2	0.634
Intellectual activities	2371.83	1.81	2	0.406
Loneliness	2374.32	2.49	2	0.289
Stroke	2377.70	3.38	2	0.185
Quality of life (QoL)	2381.90	4.21	2	0.122
Alcohol use	2386.23	4.33	2	0.115
Included in the final model				
Gender	2391.97	5.74	2	0.057
Educational level	2410.31	24.07	12	0.020
Age	2393.12	6.88	2	0.032
Depression	2413.04	26.81	2	<0.001
Social activities	2395.54	9.31	2	0.010
Social network	2393.95	7.72	2	0.021
Immediate recall	2391.35	5.12	2	0.077
Delayed recall	2442.02	55.79	2	<0.001
Orientation	2401.56	15.33	2	<0.001
Numeracy	2395.11	8.87	2	0.012
Verbal fluency	2451.25	65.02	2	<0. 001

**Table 3:** Parameter estimates from the multinomial logistic regression.

Predictors	Odds Ratio (95% CI)	
	MCI	SCI
Gender (reference: female)	0.81 (0.63–1.04)	0.63 * (0.42–0.96)
Age	0.99 (0.97–1.01)	1.03 (0.99–1.06)
Educational Level (ISCED-97 coding)		
None	0.62 (0.13–2.84)	0.13 (0.01–1.93)
Primary education (code 1)	0.58 (0.14–2.36)	0.35 (0.03–4.36)
Lower secondary education (code 2)	1.16 (0.29–4.67)	0.58 (0.05–7.25)
Upper secondary education (code 3)	0.95 (0.24–3.73)	0.83 (0.07–9.98)
Post-secondary education (code 4)	0.62 (0.11–3.61)	0.69 (0.03–14.94)
Tertiary education first stage (code 5)	1.04 (0.26–4.09)	0.74 (0.06–9.20)
Depression	1.15 * (1.08–1.22)	1.19 * (1.09–1.31)
Social activity participation	0.79 * (0.68–0.92)	0.95 (0.71–1.26)
Social network	1.05 * (1.01–1.09)	1.05 (0.99–1.11)
Immediate recall	0.88 * (0.79–0.99)	0.91 (0.76–1.08)
Delayed recall	0.80 * (0.73–0.87)	0.60 * (0.51–0.70)
Orientation	0.99 (0.73–1.34)	0.54 * (0.37–0.77)
Numeracy	0.90 * (0.82–0.99)	0.81 * (0.71–0.94)
Verbal fluency	0.95 * (0.93–0.97)	0.85 * (0.81–0.89)

Notes: the reference category of the outcome variable is normal cognitive function; * *p* < 0.05; MCI = mild cognitive impairment; SCI = severe cognitive impairment; CI = Confidence Interval.

## Data Availability

The data that support the findings of this study are available to the entire research community free of charge from the SHARE Research Data Center (www.share-project.org). Restrictions apply to the availability of these data, which were used under license for the current study; therefore, the data are not publicly available. The data are, however, available from the authors upon reasonable request and with permission of the SHARE Project (https://share-eric.eu/data/data-access (accessed on 20 October 2025)).

## Abbreviations

The following abbreviations are used in this manuscript:

SA	Successful Ageing
SOC	Selection Optimization and Compensation (SOC)
Comprehensive PCP	Comprehensive Preventive Corrective Proactive
QoL	Quality of Life
MCI	Mild Cognitive Impairment
AD	Alzheimer’s Disease
SCI	Severe Cognitive Impairment
SHARE	Survey of Health, Ageing and Retirement in Europe
SHARE-HCAP	Survey of Health, Ageing and Retirement in Europe and Harmonized Cognitive Assessment Protocol association
ISCED-97	International Standard Classification of Education
